# Magnetic Resonance Imaging Preprocessing for Robust Spinal Cord Segmentation in Cervical Myelopathy

**DOI:** 10.3390/jimaging12070323

**Published:** 2026-07-17

**Authors:** Hediyeh Toufani, Richard M. Dansereau, Philippe Phan, Jefferson R. Wilson, Eve C. Tsai

**Affiliations:** 1 Department of Mechanical Engineering, University of Ottawa, Ottawa, ON K1N 1A2, Canada; h.toufani@uottawa.ca; 2 Neuroscience Program, Ottawa Hospital Research Institute, The Ottawa Hospital, Ottawa, ON K1Y 4E9, Canada; 3 Department of Systems and Computer Engineering, Carleton University, Ottawa, ON K1S 5B6, Canada; rdanse@sce.carleton.ca; 4 Division of Orthopedics, Department of Surgery, Faculty of Medicine, University of Ottawa, Ottawa, ON K1H 8M5, Canada; pphan@toh.ca; 5 Division of Neurosurgery, Department of Surgery, University of Toronto, Toronto, ON M5S 1A1, Canada; jefferson.wilson@unityhealth.to; 6 Division of Neurosurgery, Department of Surgery, Faculty of Medicine, University of Ottawa, Ottawa, ON K1H 8M5, Canada

**Keywords:** spinal cord MRI, cervical myelopathy, deep learning segmentation, pathology-aware preprocessing, multi-representation input, boundary enhancement

## Abstract

Accurate spinal cord segmentation is important for quantitative analysis of spinal cord magnetic resonance imaging, including measurement of cross-sectional area and diffusion-based microstructural characterization. In pathological conditions like cervical myelopathy, the shape deformation induced by cord compression is extreme, rendering automated segmentation particularly challenging. While deep learning-based methods yield good results in healthy or mildly pathological cases, their reliability suffers when anatomical assumptions fail under compression. In this work, we introduce a pathology-aware, boundary-focused preprocessing framework that directly aims to mitigate failure modes imposed by cord compression. Instead of generic preprocessing, each component aims to enhance intensity homogeneity, suppress noise and improve boundary visibility. At the core of this approach is a multi-representation input derived from a single T2*-weighted scan, whereby complementary intensity-, contrast- and edge-enhanced representations are fed to the U-Net model. The proposed framework is evaluated on spinal cord MRI data from three clinical centers (194 cervical myelopathy cases). The results demonstrate that the proposed preprocessing framework improves segmentation accuracy, robustness, and stability, particularly in anatomically challenging regions affected by compression. These findings highlight the importance of pathology-aware preprocessing for reliable spinal cord segmentation in cervical myelopathy.

## 1. Introduction

The accurate segmentation of the spinal cord (SC) is a prerequisite for quantitative SC magnetic resonance imaging (MRI) analysis, such as cross-sectional area estimation, diffusion tensor imaging (DTI) metrics, and tractography. Among these applications, accurate SC segmentation is particularly important for diffusion-based analyses, as SC masks are used to define the anatomical region from which DTI metrics are extracted and within which tractography is performed. Consequently, segmentation errors directly propagate into measurements of fractional anisotropy (FA), mean diffusivity (MD), axial diffusivity (AD), radial diffusivity (RD), and tractography-derived structural features. These downstream measurements are highly sensitive to segmentation accuracy, where even minor boundary inaccuracies can lead to substantial quantitative errors. Since DTI and tractography have been extensively investigated as imaging biomarkers of SC microstructural injury and disease severity in cervical myelopathy, robust SC segmentation is essential for obtaining reliable and clinically meaningful quantitative MRI measurements [1].

Existing SC segmentation approaches can be broadly categorized into three groups: conventional image-processing methods [2,3,4,5,6,7,8], atlas-based techniques [9], and deep learning-based methods [10,11,12,13,14]. Conventional approaches typically rely on intensity thresholding [2], edge detection [3], active contours [4,5,6], or deformable models [7] to identify SC boundaries. While these methods can perform adequately in healthy subjects, they are highly sensitive to image noise, intensity inhomogeneity, and weak tissue boundaries [15]. Atlas-based methods improve robustness by incorporating anatomical priors through image registration; however, their performance depends on the assumption of relatively preserved SC anatomy and may deteriorate in the presence of severe compression and anatomical deformation [16].

More recently, deep learning approaches, particularly U-Net-based architectures and transformer-enhanced segmentation networks, have achieved state-of-the-art performance in SC segmentation [10,11,12,13,14]. These methods learn complex image features directly from annotated data and generally outperform traditional techniques. Nevertheless, most existing models have been developed and validated on healthy subjects or diseases such as multiple sclerosis [7,11,12], where the SC maintains a relatively regular morphology and is surrounded by a clearly visible cerebrospinal fluid (CSF) boundary. In cervical myelopathy, severe SC compression often causes local deformation, reduced SC-CSF contrast, partial disappearance of the CSF boundary, image noise amplification, and increased intensity variability across patients and imaging protocols [17]. As a result, even advanced deep learning models frequently experience substantial performance degradation in compressed regions [18].

Most previous studies have primarily focused on developing more sophisticated network architectures to improve segmentation accuracy [18]. In contrast, relatively limited attention has been given to the role of image preprocessing, despite the fact that many of the challenges associated with compressed SC MRI originate from image quality and boundary visibility rather than network design alone.

Preprocessing is thus necessarily a key design aspect of medical imaging pipelines, being routinely performed in order to regularize inputs prior to analysis via many operations (e.g., cropping, resampling, intensity normalization) [7]. Although much of the early deep learning literature took an opposing view—that preprocessing may not be necessary, assuming that sufficiently expressive models can learn to internalize compensation for acquisition-related variability [19]—increasing evidence is emerging that targeted preprocessing has significant benefits on robustness, generalization and clinical reliability. This is especially valuable in scenarios where the datasets are limited, image quality is degraded, or anatomical structures differ from standard expectations [20].

**Figure 1:**
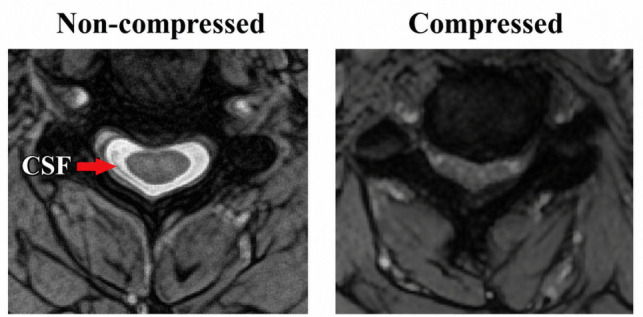
Axial SC T2* Weighted MRI in non-compressed (**left**) and compressed (**right**) conditions, showing reduced boundary contrast under compression.

Cervical myelopathy resulting from cervical stenosis leads to compression of the SC, altering its normal cylindrical geometry. In T2*-weighted MRI, this compression often causes the SC to appear flattened or deformed—commonly described as pancake- or trefoil-shaped—rather than maintaining its typical circular or oval cross-section. In many cases, the stenosis is asymmetric or non-uniform, resulting in uneven deformation where one side of the cord is more compressed than the other. In addition, the surrounding CSF, which normally provides strong boundary contrast, may be displaced or partially obliterated, further reducing the visibility of cord boundaries [21,22] (Figure 1). This leads to at least three compounding challenges for segmentation algorithms: loss of contrast and addition of noise, substantial variability across scanners and acquisition protocols, and collapse of the cord–CSF boundary [14,23]. Existing pipelines [7,11,12] generally apply preprocessing in a uniform, task-agnostic manner or delegate the workload to the segmentation network, which must learn robustness implicitly. Under severe compression, these assumptions often fail, leading to boundary leakage, under-segmentation, and poor generalization across sites.

These limitations underlie the demand for preprocessing techniques that directly consider pathological deformation and degradation of boundaries. In this work, we contend that compressed SC MRI represents a unique imaging regime that requires pathology-aware preprocessing. Instead of complicating the architecture, we propose a boundary-focused preprocessing mechanism that naturally transforms the learning problem. The major innovation of this work is to define a multi-representation input from a single MRI modality, in which complementary views of intensity, local contrast, and edge structure are provided jointly as inputs to the network. This strategy inherently embeds boundary priors without strong shape penalties, which ideally fits anatomically distorted cases. By reinforcing boundary information lost under compression, the proposed approach improves segmentation accuracy, robustness, and stability across SC MRI data from multiple clinical sites.

## 2. Materials and Methods

### 2.1. Dataset and Imaging Strategy

This study includes SC MRI data from three independent clinical sites (Site 1: *n* = 60, Site 2: *n* = 69, Site 3: *n* = 65), for a total of 194 cases. Although all subjects in the dataset were diagnosed with cervical myelopathy associated with cervical stenosis, SC compression typically affected only a limited number of axial slices along the cord. In most MRI volumes, the majority of slices showed relatively normal SC morphology, while compression was present only at specific levels. As a result, the dataset naturally contained a mixture of normal-appearing cord structures and pathological deformation within the same scans. This diversity allowed the model to learn both typical SC anatomy and the structural variations introduced by compression, which supports model robustness and generalization.

Data were retrospectively collected, fully anonymized, and approved by the institutional research ethics boards at the participating sites.

Imaging protocols vary across sites in terms of scanner vendor, field strength, and acquisition parameters, representing real-world clinical variability. This heterogeneity naturally engendered a testbed for cross-site validation.

In this study, SC segmentation was performed on T2*-weighted MRI. This modality is fundamental in the analysis of diffusion MRI, as these images are acquired with higher spatial resolution and clearer anatomical definition at the boundary between the SC and adjacent tissues than diffusion-weighted images. Therefore, they were used to build SC masks that would be registered into diffusion space [24].

All SC MRI images from patient cases at Sites 1 and 2 were acquired using a Siemens MAGNETOM PrismaFit 3T MRI scanner (Siemens Healthineers, Erlangen, Germany), whereas those from Site 3 were obtained using a Siemens MAGNETOM Skyra 3T scanner (Siemens Healthineers, Erlangen, Germany), although acquisition parameters differed across sites. The T2*-weighted imaging parameters were as follows:

- Site 1: Slice Thickness: 3.99 mm, Field of View (FOV): [180, 180, 96] mm, Image Size: [320, 320, 24] (X, Y, Z) voxels, Voxel Size: 0.56 × 0.56 × 3.99 mm3.
- Site 2: Slice Thickness: 3.99 mm, FOV: [200, 150, 56] mm, Image Size: [256, 192, 14] (X, Y, Z) voxels, Voxel Size: 0.78 × 0.78 × 3.99 mm^3^.
- Site 3: Slice Thickness: 3.3 mm, FOV: [180, 180, 125] mm, Image Size: [512, 512, 38] (X, Y, Z) voxels, Voxel Size: 0.35 × 0.35 × 3.3 mm^3^.

To assess the distribution of SC compression across the dataset, all axial slices were grouped based on their compression ratio (CR), which was computed from the manually annotated ground-truth segmentation masks. The CR was defined as the ratio of the anteroposterior (AP) diameter to the transverse diameter of the SC at its point of maximum compression [25], given as

(1) $$CR=\frac{AP\;diameter}{Transverse\;diameter}$$

Based on this ratio, slices were categorized as non-compressed (CR ≥ 0.80), moderately compressed (0.40 ≤ CR < 0.80), or severely compressed (CR < 0.40) [25].

Table 1 summarizes the distribution of slice categories in the training and test datasets. Similar distributions between the training and test sets were maintained to minimize sampling bias.

Before applying the investigated preprocessing techniques, all MRI volumes underwent two essential standardization steps: resampling and central cropping. Because the datasets were acquired at different institutions using different scanners and imaging protocols, the original images exhibited substantial variability in voxel spacing, FOV, and image dimensions. To ensure consistency across all datasets, each volume was first resampled to a common spatial resolution of 0.35 × 0.35 × 3.3 mm^3^. Subsequently, a fixed-size central crop of 128 × 128 × 15 voxels centered on the SC region was applied to all volumes. These steps standardized the spatial characteristics of the images, reduced inter-site variability, and ensured that anatomically corresponding structures occupied comparable image locations and scales across all subjects. As a result, images from different sites became directly comparable and could be processed using identical preprocessing parameters and network architectures.

### 2.2. Boundary-Focused, Pathology-Aware Preprocessing

Pre-processing of the input data was designed to address three failure modes: (1) intensity inhomogeneity and noise, (2) variability across imaging sites and devices, and (3) loss of contrast at the boundary between the cord and surrounding tissue due to compression. Each component then addresses one of these challenges, and its impact is assessed.

#### 2.2.1. Intensity Inhomogeneity Correction and Denoising

T2*-weighted SC MRI often suffers from intensity inhomogeneity (bias field) and noise, both of which degrade boundary definition and reduce segmentation quality [26,27]. Various strategies have been proposed to address these issues, including bias field correction, denoising, spatially adaptive filtering, tissue- or ROI-specific normalization, B1 field mapping, and more recent deep-learning-based harmonization approaches. In this work, we focused on bias field correction and denoising, as they represented widely used and computationally efficient preprocessing steps for improving intensity homogeneity and boundary visibility.

##### Bias Field Correction

The bias field inhomogeneity is characterized by smooth, low-frequency intensity shading due to non-uniform RF coil sensitivity, B1 field imperfections, and subject positioning. In T2*-weighted SC MRI, it can yield:

- Inconsistent intensity across slices;
- Artificial bright/dark regions unrelated to anatomy;
- Reduced separability between cord tissue and surrounding structures [28].

We applied 3D N4 bias field correction [29] to correct for low-frequency intensity inhomogeneity present in SC MRI. N4 bias field correction was selected because it provides robust correction of low-frequency intensity inhomogeneity while preserving anatomical structures, making it particularly suitable for SC MRI datasets acquired across multiple sites and imaging protocols. The observed image was modelled as the product of the true tissue intensity and a smoothly varying bias field, with additional noise. N4 estimates this bias field as an additive process rather than a multiplicative one by applying logarithmic intensity mapping in order to provide a fast estimate of bias. The bias field was parameterized using low-resolution B-spline basis functions and iteratively refined using an entropy-minimization framework that sharpens tissue intensity distributions. After estimation, the bias field was exponentiated and removed from the original image to recover the corrected tissue intensities [29].

In this study, N4 was performed in 3D over the entire image volume to enable spatially consistent correction across slices. The 3D configuration reduces slice-wise artifacts and anatomical discontinuity and generates more homogeneous intensity profiles that optimize SC segmentation fidelity.

##### Denoising

To suppress image noise while preserving anatomical structures, NLM denoising [30] was applied to all SC MRI volumes. NLM denoising was selected because it suppresses random image noise while preserving fine anatomical structures and boundary information [30], which are critical for accurate segmentation of compressed SC regions.

NLM estimates the intensity of each voxel using a weighted average of voxels with similar image neighbourhoods, thereby reducing random noise while preserving structural boundaries. The denoising procedure was applied using identical parameters across all datasets.

NLM denoising was applied using a patch radius of 1 voxel and a block radius of 3 voxels. For each MRI volume, the noise level was estimated independently using an automatic noise estimation procedure, and the resulting noise variance was used to guide the denoising process. A Rician noise model was assumed during denoising, which is appropriate for magnitude MRI data. By estimating the noise level separately for each volume, the denoising strength was adaptively adjusted according to the noise characteristics of the individual image while preserving anatomical boundaries and fine structural details.

#### 2.2.2. Mitigating Multi-Site Variability

All types of clinical datasets are usually subject to variations for different scanner manufacturers, field strength, pulse sequence parameters, resolution, reconstruction scaling, and coil configuration. These shifts may create intensity distribution changes and texture differences, leading to reduced model performance when training and testing distributions do not match [31]. We tackle this using intensity normalization and augmentation.

##### Normalization

Z-score normalization standardizes voxel intensities by subtracting the mean and dividing by the standard deviation, yielding approximately zero mean and unit variance per image (or defined region of interest (ROI)), which reduces dependence on absolute intensity scaling [32,33]. In the field of medical imaging, this leads to improved cross-subject comparability and stabilizes model optimization, leading to better generalization across acquisition conditions [34].

As the pipeline cuts to a central region around the SC very early in preprocessing, large areas of background and irrelevant anatomy are removed. This leads to reduced biological variability from adjacent structures (vertebrae, muscle and fat), with inter-subject variability then being dominated by scanner-dependent voxel intensity scaling. Normalization is thus particularly powerful, dampening differences in contrast inherent to the scanner but preserving structural information needed for segmentation.

Z-score normalization standardizes the intensity distribution in each image by subtracting the mean and dividing by the standard deviation:

(2) $$I_{norm}\left(x\right)=\frac{I\left(x\right)-\mu }{\sigma }$$

where μ and σ identify the mean and standard deviation of non-background voxels in the image [33].

This transformation standardizes each volume to approximately zero mean and unit variance while preserving the relative intensity relationships and spatial structure of the image. Consequently, anatomical boundaries and tissue contrast patterns are retained, and only the numerical intensity scale is modified, minimizing scanner-related intensity differences without introducing loss of structural information relevant to segmentation.

##### Data Augmentation

To reduce overfitting and improve robustness to acquisition and positioning variability, we applied the following augmentations during training:

These augmentations are particularly applicable for SC MRI, where both patient positioning differences and pathological distortion arise in typical clinical data [35].

- Intensity transformations: Random intensity scaling within a factor range of 0.9–1.1, random intensity shifting within ±0.1 of the normalized intensity range, and Gaussian noise injection (σ = 0.01) to simulate variability in image contrast and noise levels across scanners and acquisition protocols.
- Geometric transformations: Random rotations within ±10° were applied only in the axial plane, translations of up to ±10 voxels in the axial plane, and scaling within a range of 0.9–1.1 to improve robustness to differences in patient positioning, SC orientation, and anatomical size.
- Elastic deformation: Smooth displacement fields with a maximum displacement of 2 voxels and Gaussian smoothing to mimic realistic anatomical variability and mild tissue deformation while preserving the overall SC anatomy.

The same geometric transformations were applied simultaneously to the MRI volume and its corresponding ground-truth segmentation mask.

#### 2.2.3. Boundary Contrast Restoration Under Cord Compression

A hallmark pathology in myelopathy is boundary contrast loss at the cord margin. Under normal conditions, CSF provides a bright rim that separates the cord from the surrounding tissue. In compressed regions, CSF can be diminished or non-existent, and the cord may come into contact with similarly dense tissue, creating blunted or missing edges (Figure 1), which impair both classical and deep-learning-based segmentation [21].

While the majority of previous work on SC preprocessing has focused on denoising and normalization [14,23], significantly less attention has been given to directly combating loss of contrast at boundaries due to compression. To address this, we introduce a three-part strategy: (i) local contrast enhancement, (ii) edge-focused filtering, and (iii) multi-channel input design in order to expose complementary cues to the network.

##### Local Contrast Enhancement

**Figure 2:**
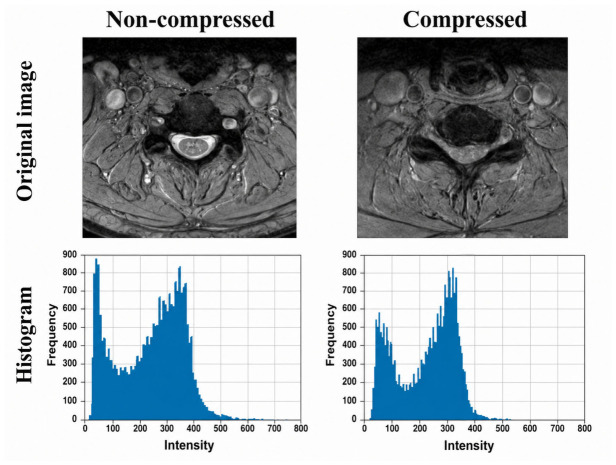
Comparison of intensity histograms between a non-compressed and a compressed SC slice.

In general, the intensity distribution of a non-compressed slice is more dispersed and contains high-intensity (bright) pixels correlated to CSF around SC (see Figure 2). On the contrary, the compressed slice does not have bright pixel values, and nearly all intensities are concentrated in a lower range.

The observed differences between the histograms do not indicate information loss caused by preprocessing. Rather, they reflect the underlying anatomical and contrast changes associated with SC compression. In non-compressed slices, high-intensity voxels originating from the surrounding CSF contribute to a broader intensity distribution, whereas compressed slices exhibit a narrower distribution due to partial loss of the SC-CSF interface. This reduction in boundary-related intensity information motivates the use of normalization, contrast enhancement, and edge enhancement techniques in the proposed preprocessing pipeline to improve boundary visibility prior to segmentation.

Contrast-limited adaptive histogram equalization (CLAHE) [36] was applied to all SC MRI volumes to enhance local image contrast and improve boundary visibility. CLAHE was selected because it enhances local contrast while limiting excessive noise amplification, making it well-suited for improving SC boundary visibility in regions affected by compression. The algorithm divides the image into small local regions (tiles) and performs histogram equalization independently within each region. To prevent excessive enhancement of noise, the local histogram is clipped at a predefined threshold (clip limit) before redistribution of the clipped intensities. The enhanced regions are subsequently combined using interpolation to avoid artificial intensity discontinuities between neighbouring tiles. The algorithm performs histogram equalization within local image regions while limiting contrast amplification through histogram clipping, thereby reducing the risk of noise enhancement. CLAHE was applied using identical parameters across all datasets.

CLAHE was applied after Z-score normalization using fixed parameters for all images. No dynamic adjustment of the clip limit was performed, as intensity standardization produced comparable intensity distributions across subjects and acquisition sites.

##### Edge Enhancement

To enhance SC boundary visibility, Laplacian-of-Gaussian (LoG) [37] filtering was applied to all MRI volumes. LoG combines Gaussian smoothing with Laplacian edge detection to generate an edge-enhanced image representation while reducing sensitivity to image noise. This approach was selected because compressed SC regions often exhibit poorly defined SC-CSF boundaries, making boundary information difficult to identify in the original image. Rather than serving as an additional denoising step, the LoG image was used as a complementary representation to emphasize SC contours and provide boundary-focused information for the segmentation network.

The LoG operator introduces a combination of Gaussian smoothing with the second-order derivative (Laplacian) operation. The first Gaussian smoothing reduces high-frequency noise, while the second Laplacian highlights locations of rapid intensity change with respect to their surroundings, which correspond to anatomical features. This formulation enables selective amplification of edge structures while avoiding the noise sensitivity that comes with pure derivative operators. As a result, this enhancement of cord boundaries helps address the subtle cord boundaries that are difficult to differentiate between raw and smoothed images.

Importantly, this step is not intended to do any denoising or replace earlier noise reduction steps. Instead, it outputs an edge-enhanced representation that explicitly highlights the SC contour, particularly in compressed regions where the cord–CSF boundary is poorly defined. This representation is utilized as a complementary input for the segmentation network, providing boundary-specific hints to achieve both more accurate and more stable segmentation [37].

LoG filtering was applied using a fixed Gaussian scale parameter of σ = 1.0 voxel. This value was selected to balance edge enhancement and noise suppression. Larger sigma values produced excessive smoothing of SC boundaries, whereas smaller values increased sensitivity to image noise and generated unstable edge responses. Because all images were resampled to a common spatial resolution prior to preprocessing, the same sigma value was applied consistently across all datasets.

##### Multi-Channel Inputs

MRI data analysis is typically single-channel, which limits the diversity of input cues available to the network. To improve the input representation, we used a multi-channel approach with multiple representations of a single T2* scan. In particular, three complementary channels were constructed from the same T2* image:

- Channel 1: Raw T2*: preserves native anatomical context
- Channel 2: CLAHE-enhanced T2*: restores local contrast in regions without CSF
- Channel 3: LoG edge-enhanced T2* emphasizes edge and gradient information at weak boundaries
- This is expressed as:

(3) $$X=[X_{Raw},X_{CLAHE},X_{Laplacian}]$$

where $X_{Raw}$ represents the original SC MRI image after the preceding preprocessing steps (N4 bias field correction, NLM denoising, and Z-score normalization), $X_{CLAHE}$ represents the contrast-enhanced image generated using CLAHE, and $X_{Laplacian}$ represents the edge-enhanced image produced using LoG filtering. These three representations are concatenated along the channel dimension to form the final multi-channel input supplied to the segmentation network.

The original, CLAHE-enhanced, and LoG-enhanced images were concatenated along the channel dimension to form a three-channel input. This representation was provided directly to the segmentation network, enabling early fusion of intensity, contrast, and edge information through the first convolutional layer.

##### Data Leakage Prevention

To prevent data leakage in the cross-site experiments, all preprocessing operations were performed independently for each volume. N4 bias field correction estimated the bias field separately for each image, NLM denoising estimated noise characteristics independently for each volume, and Z-score normalization used only the intensity statistics of the individual image being processed. No site-level or dataset-level statistics were used during preprocessing, and no information from the test set was incorporated into the training pipeline.

In addition, each of the 194 cases corresponded to a single MRI volume from a unique patient. Consequently, the dataset was split at the patient level, ensuring that no patient contributed data to more than one of the training, validation, or test subsets, thereby eliminating patient-level data leakage.

### 2.3. Experimental Design

The experimental framework was designed to systematically evaluate the impact of each preprocessing step on segmentation performance. The pipeline was designed sequentially and cumulatively, rather than applying all preprocessing operations at once. Each preprocessing component was introduced one at a time and added to the previously applied steps. The experimental design is summarized in Table 2.

**Figure 3:**
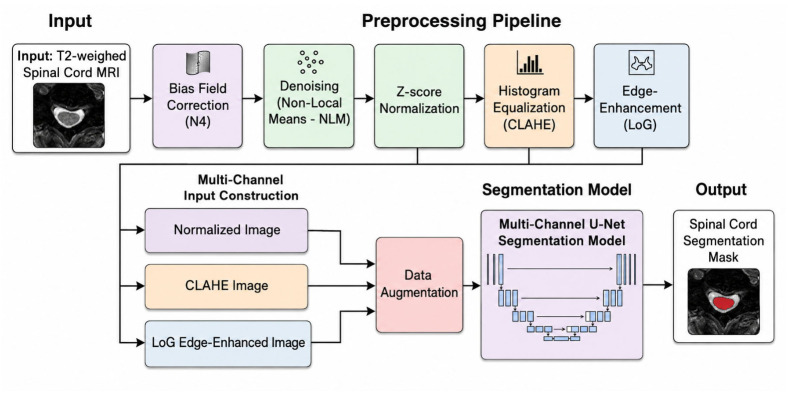
Overview of the proposed preprocessing and segmentation pipeline. T2*-weighted SC MRI images undergo a series of preprocessing operations, including N4 bias field correction, non-local means denoising, Z-score normalization, data augmentation, contrast enhancement using CLAHE, and edge enhancement using LoG. The original, CLAHE-enhanced, and LoG-enhanced images are combined to form a multi-channel input, which is subsequently processed by a U-Net-based segmentation model to generate the final SC segmentation mask. The figure illustrates the complete preprocessing pipeline used in the final configuration. For the experimental comparison of preprocessing strategies, each configuration was evaluated independently by training a newly initialized model from scratch using identical training, validation, and test datasets.

Figure 3 illustrates the proposed preprocessing pipeline applied to T2*-weighted SC MRI images prior to segmentation. The image undergoes a series of preprocessing operations designed to reduce inter-subject and inter-site variability, improve image quality, and enhance SC boundary visibility. These preprocessing operations are applied sequentially, with the output of each step serving as the input to the subsequent step. The resulting representations, including the original image, CLAHE-enhanced image, and LoG-enhanced image, are combined to form a multi-channel input that is subsequently provided to the segmentation model to generate the final SC mask. It should be noted that Figure 3 depicts the complete preprocessing workflow only. During the experimental evaluation, each cumulative preprocessing configuration was treated as an independent experiment, and a newly initialized segmentation model was trained from scratch using identical training, validation, and test datasets.

The MONAI [38] 3D U-Net was selected due to its robustness, reproducibility, and widespread acceptance in segmentation tasks for medical images, including SC MRI. The network was configured with five resolution levels and channel sizes of [16, 32, 64, 128, 256]. Down-sampling was performed using strided convolutions with strides of (2, 2, 2) at each resolution level, while up-sampling was implemented through transposed convolutions. All convolutional layers had a kernel size of 3 × 3 × 3 and were followed by instance normalization and ReLU activation. Skip connections were implemented as direct feature map concatenation between corresponding encoder and decoder levels, without additional processing layers.

The input consisted of either a single channel or three channels, depending on the preprocessing configuration, while the output was a single-channel binary SC segmentation mask.

The network architecture and all the training hyperparameters were kept constant across all experiments, to allow for fair comparison of the various preprocessing strategies. The model was trained using a Dice loss function that directly optimized spatial overlap between predicted and ground-truth masks. The Adam optimizer (learning rate, 1 × 10^−4^; β_1_ = 0.9; β_2_ = 0.999) was used for optimization. A batch size of 4 was fixed across all the experiments. Training was performed for a fixed number of epochs with early stopping based on the validation Dice score. Note that there was no architectural tuning or hyperparameter optimization applied.

The complete dataset comprising 194 cases was randomly shuffled and split into training (70%, *n* = 135), validation (15%, *n* = 29), and test (15%, *n* = 30) subsets. The split was performed across the pooled dataset containing cases from all three clinical sites. Consequently, each subset included cases from all participating sites. This study therefore evaluates generalization under a random multi-site split rather than a site-based holdout design.

All experiments were implemented in Python 3.9.6 using the PyTorch 2.2.1 and MONAI 1.3.0 frameworks. Model training was performed on a MacBook Pro equipped with an Apple M1 processor (Apple Inc., Cupertino, CA, USA). Each preprocessing configuration was trained independently from scratch, and the average training time for a single model was approximately 1 h and 20 min, including validation and early stopping.

### 2.4. Image Quality Assessment

To quantitatively evaluate the effects of the proposed preprocessing techniques, several image quality metrics were computed, including the coefficient of variation (CoV), Signal-to-Noise Ratio (SNR), contrast-to-noise ratio (CNR), inter-subject intensity variance, and edge preservation index (EPI).

To evaluate the effectiveness of N4 bias field correction in improving intensity homogeneity within the SC, the CoV was calculated using the manually segmented SC mask as the ROI. CoV was computed as

(4) $$CoV=\frac{\sigma_{SC}}{\mu_{SC}}$$

where $\mu_{SC}$ and $\sigma_{SC}$ denote the mean and standard deviation of voxel intensities within the SC ROI, respectively. Lower CoV values indicate greater intensity homogeneity and therefore more effective correction of intensity inhomogeneity.

To evaluate the effectiveness of NLM denoising in suppressing noise while preserving the image signal, the SNR was calculated. The manually segmented SC mask was used as the SC ROI, and background noise was estimated from the corner regions of the original MRI volume. SNR was computed as

(5) $$SNR=\frac{\mu_{SC}}{\sigma_{noise}}$$

where $\mu_{SC}$ is the mean SC intensity, and $\sigma_{noise}$ is the standard deviation of the background noise ROI. Higher SNR values indicate improved image quality through noise reduction while preserving the underlying anatomical signal.

To assess the effectiveness of NLM denoising and CLAHE enhancement in improving the separation between the SC and surrounding tissues, the CNR was calculated as

(6) $$CNR=\frac{{|\mu }_{SC}-\mu_{surround}|\;}{\sigma_{noise}}$$

where $\mu_{SC}$ is the mean intensity within the SC ROI, $\mu_{surround}$ is the mean intensity within the surrounding-tissue ring ROI, and $\sigma_{noise}$ is the standard deviation of background noise estimated from corner regions of the original MRI volume. A surrounding-tissue ROI was generated by dilating the SC mask to create a ring surrounding the cord while excluding background voxels. Higher CNR values indicate improved contrast and greater visibility of SC boundaries.

To evaluate the effectiveness of Z-score normalization in reducing scanner- and protocol-related intensity variability, inter-subject intensity variance was computed across all subjects before and after normalization. For each subject, the mean image intensity ($\mu_{i}$) was calculated, and the variance across subjects was determined as

(7) $${Var}_{inter}\frac{1}{N}\sum_{i=1}^{N}(\mu_{i}-\bar{\mu }{)}^{2}$$

where N denotes the number of subjects, $\mu_{i}$ is the mean image intensity of subject *i* and $\bar{\mu }$ is the average intensity across all subjects. Lower inter-subject variance indicates greater consistency of intensity distributions across subjects and acquisition sites.

To evaluate the effectiveness of LoG filtering in preserving and enhancing anatomical boundary information, the EPI was computed [39]. EPI quantifies the extent to which edge information is retained following preprocessing and was calculated as

(8) $$EPI=\frac{\sum \left|\nabla I_{LoG}\right|}{\sum \left|\nabla I_{original}\right|}$$

where $I_{original}$ and $I_{LoG}$ denote the original and LoG-enhanced images, respectively, and ∇ represents the image gradient operator. EPI values closer to 1 indicate better preservation of anatomical edge information after preprocessing [39].

### 2.5. Segmentation Evaluation Metrics

For every preprocessing configuration, all models were trained from scratch using the same training, validation, and test splits. Since the proposed framework performs segmentation on complete 3D MRI volumes, model training and inference were conducted at the volumetric level. Segmentation performance was evaluated using the Dice similarity coefficient (Dice), precision, and recall [15].

To assess the impact of SC compression on segmentation performance, evaluation was performed at both the volume and slice levels. Volumetric metrics were computed over the entire SC volume to quantify overall segmentation accuracy. In addition, a slice-level analysis was performed to investigate performance across different degrees of compression.

Slice-wise Dice scores were subsequently calculated and reported separately for each compression category. This evaluation strategy enabled assessment of overall segmentation performance while also quantifying the effect of compression severity on segmentation accuracy.

### 2.6. Statistical Analysis

To determine whether differences in segmentation performance between preprocessing configurations were statistically significant, pairwise comparisons of Dice scores were performed using the Wilcoxon signed-rank test. This non-parametric test was selected because Dice scores may not follow a normal distribution, and measurements were paired across the same test subjects.

For each preprocessing configuration, Dice scores were computed on the identical held-out test cases. Comparisons were performed between the baseline model and each preprocessing configuration. Statistical significance was defined as *p* < 0.05.

## 3. Results

### 3.1. Image-Quality Metrics

C1-A: Bias field correction using 3D N4.

**Figure 4:**
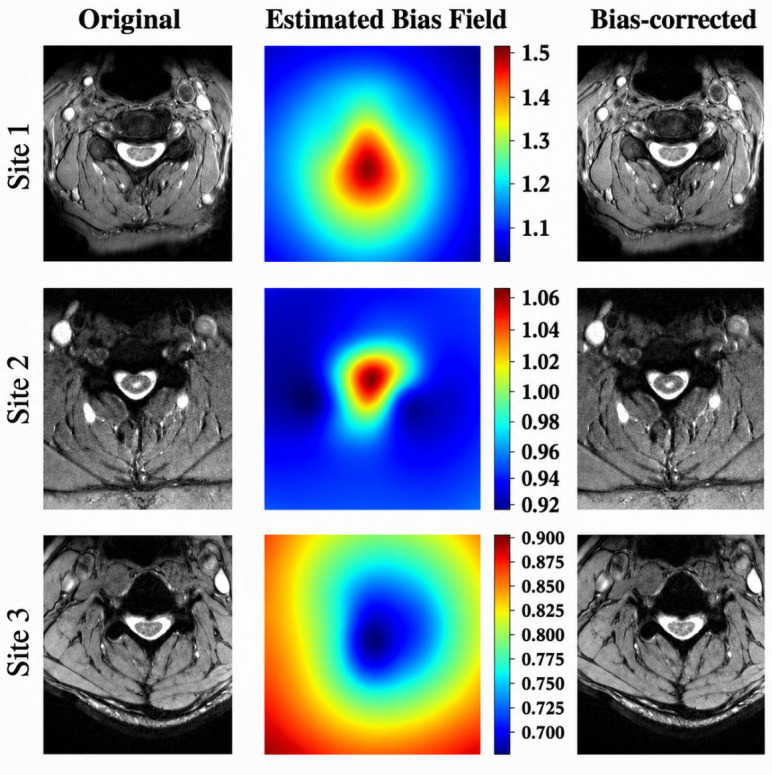
Examples of bias field correction using N4 from SC T2*-weighted images across three sites.

Bias field correction using N4 significantly mitigated intensity inhomogeneity throughout all datasets, as shown in Figure 4. The estimated bias fields were overall smooth and low-frequency, and images acquired from different sites showed site-specific patterns of spatial variability consistent with scanner- and acquisition-dependent intensity non-uniformities. After correction, these images demonstrated improved intensity homogeneity within the SC and surrounding tissues that appeared to diminish artificial intensity gradients noted in the original image.

The impact of bias field correction was quantitatively assessed using the CoV of intensities within the SC ROI before and after correction (Table 3).

As shown in Table 3, the implementation of 3D N4 yielded a stable decrease in intensity variability among the SC across all datasets. The mean CoV improved from 0.138 to 0.107 over all volumes assessed. These improvements were consistent across all three acquisition sites, which translated to improved intensity homogeneity and higher inter-subject consistency before segmentation.

C1-B: Denoising using NLM.

The denoising was quantitatively evaluated by computing the SNR and CNR between SC and CSF regions using a background ROI as noise estimate (Table 4). These metrics assess the degree of noise suppression while preserving anatomical integrity. The noise level was estimated as the standard deviation of intensities within a manually selected background window located outside the body, assumed to contain no true signal. We note that, in magnitude MRI with sum-of-squares reconstruction, background noise follows a Rayleigh distribution, which leads to an underestimation of absolute SNR. No analytical correction was applied, as SNR and CNR were used for relative comparison across preprocessing steps. Therefore, these metrics should be interpreted comparatively rather than as absolute quantitative measures.

As shown in Table 4, NLM denoising resulted in consistent increases in cord–CSF CNR across datasets. When evaluated on a per-subject basis, the mean relative increase in CNR was 15.5% and 26.1% for Site 1 and Site 2, respectively. These values reflect the average of individual subject-level percentage changes and therefore may not be directly inferred from the group mean values reported in the table.

For the Site 3 dataset, which had a significantly higher baseline SNR, only minimal changes were observed. The largest relative gains were observed here in datasets with lower baseline contrast, indicating that NLM is especially useful in preserving noise-induced contrast lost while preserving anatomical boundaries. Importantly, SC SNR remained stable across all cohorts, as evidenced by the negligible differences between pre- and post-denoising mean SNR values in Table 4 (absolute changes < 0.5% across all sites), indicating that the global signal characteristics were not distorted by NLM denoising.

C2-A: Intensity normalization using Z-score normalization.

All SC myelopathy cases underwent Z-score normalization to mitigate variance introduced by differences in scanner parameters. In particular, this method standardizes image intensities by transforming them to zero mean and unit variance, enabling consistent intensity scaling across subjects.

It is important to note that the first preprocessing step removed background and other non-vertebral anatomical information in all images via a concentric crop around the SC. This cropping step reduces the influence of surrounding tissues, such as muscle, fat, and bone marrow, which can introduce inter-subject intensity variability. As a result, the remaining variability in the cropped images was more strongly influenced by scanner-dependent factors, such as coil sensitivity, reconstruction scaling, and differences in acquisition protocols, although some degree of biological variability may have still persisted.

The normalization step was verified by measuring inter-subject statistics in the intensity before and after transformation. Z-score normalization was applied independently to each image, enforcing zero mean and unit variance at the individual sample level (Table 5). This confirms that normalization leads to standardized intensity scaling across subjects and sites, yielding an input space of harmonized features for downstream learning, rather than improving image contrast or noise characteristics.

C2-B: Data augmentation.

**Figure 5:**
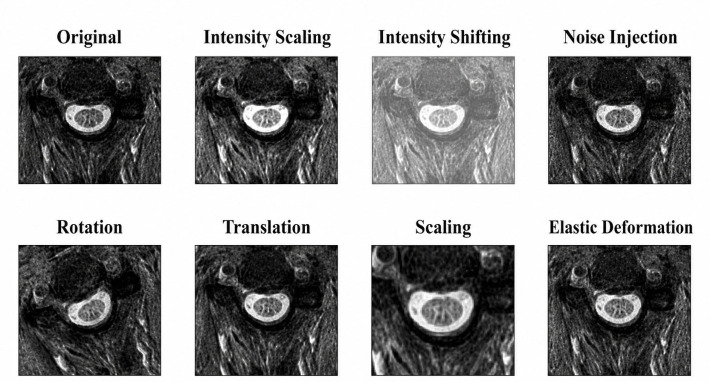
Examples of data augmentation techniques applied to an SC MRI image.

Figure 5 presents examples of the data augmentation techniques applied for training the SC MRI, including spatial, intensity-based transformations and elastic deformation. These augmentations reflect real variability in anatomy and acquisition conditions, retaining SC structure. This step does not aim to enhance image quality, but instead to improve model robustness and generalization. Thus, its effect can be seen in segmentation results through the evaluation of performance metrics.

C3-A: Local contrast enhancement using CLAHE.

**Figure 6:**
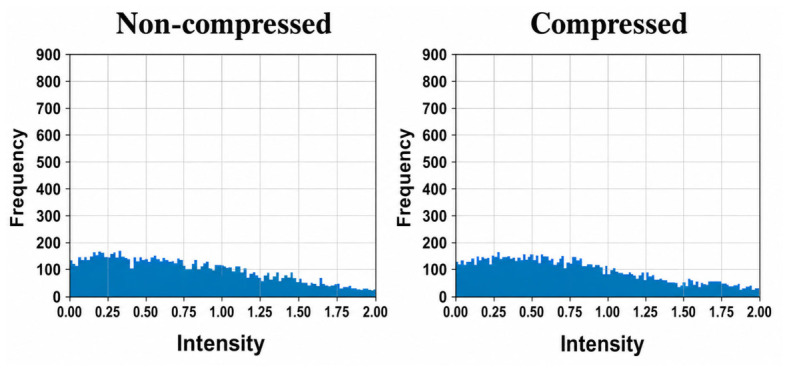
Comparison of intensity histograms between a non-compressed and a compressed SC slice after applying CLAHE.

The histograms for both a non-compressed and a compressed slice, after CLAHE processing is applied, are shown in Figure 6. Comparing Figure 6 with Figure 2, which shows the histograms of the same slices before preprocessing, shows what CLAHE does. The intensity range in Figure 6 is from 0 to 2 due to the normalization step before CLAHE.

In the uncompressed slice, a histogram of voxel intensities shows a fairly wide and smooth distribution, suggesting that tissue structures are already well separated, so CLAHE is simply providing further local contrast without causing undue distortion. The gradual spread of intensities is associated with better visualization of subtle variation within SC anatomy.

In the compressed slice, the histogram also spreads more than in the case before any enhancement (Figure 2), except this time with a clear tendency towards mid-intensity values. This indicates that CLAHE can redistribute the pixel intensities of regions where contrast was limited initially because of compression.

The quantitative assessment of the contrast enhancement was performed by computing the CNR (Table 6). Thus, these metrics evaluated the capability of the preprocessing step to improve tissue contrast without degrading the noise amplification.

Table 6 summarizes the effect of CLAHE in all sites on the CNR. CLAHE was applied for all of the groups, which resulted in increased CNR between SC and CSF. Only the Site 2 yield demonstrated the most significant improvement, climbing from 7.430 to 10.582, indicating that CLAHE might be particularly effective at improving contrast in low-contrast circumstances.

Site 1 further demonstrated a significant change (12.880–15.436), while Site 3, with its already relatively high contrast, realized more modest gains (36.193–36.947). In general, with all datasets combined, the mean CNR went from 18.752 to 21.922, demonstrating that CLAHE enhances tissue separability across heterogeneous datasets. This finding suggests that the gain in image quality from local contrast enhancement is tremendous in cases where intrinsic contrast is weak.

C3-B: Edge enhancement using LoG.

To enhance anatomical boundary information, LoG filtering was applied to all SC MRI scans. The effectiveness of this preprocessing step was evaluated using the EPI, which quantifies the proportion of edge information retained in the LoG-enhanced image relative to the original image [40]. The EPI results across all acquisition sites are summarized in Table 7.

As shown in Table 7, EPI values remained consistently high across all acquisition sites, indicating substantial preservation of anatomical edge information following LoG filtering. Site 3 achieved the highest mean EPI (0.84), followed by Site 1 (0.78) and Site 2 (0.75), with an overall mean EPI of 0.79. These findings demonstrate that LoG filtering preserved the majority of boundary information while enhancing edge representation. The consistently high EPI values suggest that LoG enhancement improves the visibility of SC contours without substantially degrading anatomical structures, thereby providing complementary boundary information for subsequent segmentation.

Visual impact of preprocessing components

**Figure 7:**
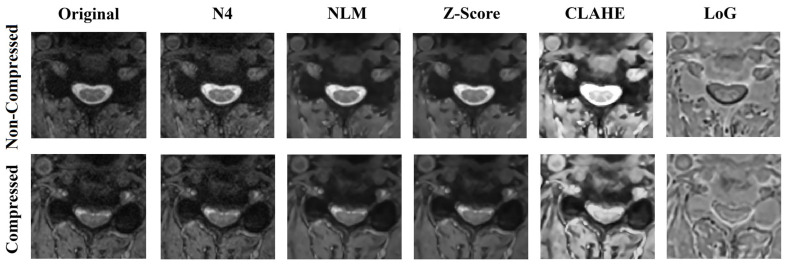
Examples of axial T2*-weighted SC MRI slices showing raw data (left) and the effect of each preprocessing technique.

Sample output for each preprocessing technique on normal and compressed SC MRI slices is shown in Figure 7.

As shown in Figure 7, N4 bias field correction improves intensity homogeneity within the cord, while NLM denoising reduces background noise without degrading anatomical structure. Z-score normalization standardizes intensity scaling across images without altering local contrast. CLAHE enhances local contrast and improves boundary visibility, particularly in compressed regions. The LoG representation emphasizes edge structures and highlights the SC contour, providing complementary boundary information for segmentation.

### 3.2. Segmentation Results

Segmentation performance across preprocessing strategies is reported in Table 8 using Dice (volume, non-compressed, moderate compression, and severe compression) precision and recall.

The baseline model achieved a mean Dice score of 81.98% at the volume level. However, slice-wise analysis revealed substantial performance differences across compression categories. Non-compressed slices achieved the highest Dice score (92.8%), whereas segmentation accuracy decreased markedly in moderately compressed (73.2%) and severely compressed slices (49.6%). These findings confirm that SC compression introduces substantial segmentation challenges beyond those observed in normal-appearing anatomy.

Single-stage preprocessing methods (C1) produced modest but consistent improvements, particularly in compressed regions. N4 bias field correction increased Dice scores from 73.2% to 75.0% in moderately compressed slices and from 49.6% to 52.5% in severely compressed slices. Similar improvements were observed following NLM denoising, suggesting that intensity homogenization and noise suppression facilitate more accurate delineation of compressed SC boundaries.

More substantial gains were observed with Z-score normalization and augmentation (C2). Augmentation improved Dice scores to 80.2% and 61.3% in moderate and severe compression, respectively, indicating enhanced robustness to anatomical variability and compression-related deformation.

The largest improvements were achieved using the boundary-focused preprocessing strategies (C3). CLAHE and LoG filtering consistently improved segmentation performance in compressed slices, supporting the importance of boundary enhancement in regions with reduced SC-to-surrounding tissue contrast. The best performance was achieved by the proposed multi-channel representation (C3-C), which combines the original, CLAHE-enhanced, and LoG-enhanced images. This configuration achieved a mean volume Dice of 86.42%, while Dice scores increased to 84.6% and 68.4% in moderately and severely compressed slices, respectively. The multi-channel approach also achieved the highest precision (87.33%) and recall (89.85%), demonstrating superior segmentation performance across all compression categories.

**Figure 8:**
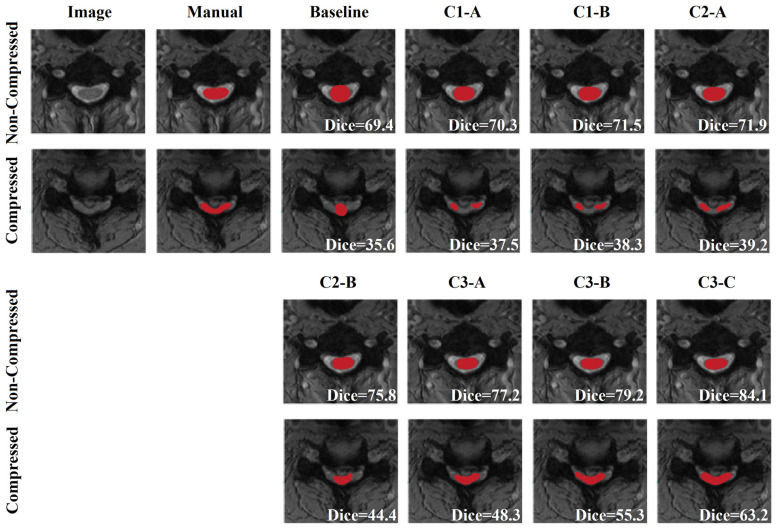
Qualitative comparison of SC cross-sectional area segmentation results after successive preprocessing stages for non-compressed and compressed cases. Dice similarity coefficient values are displayed within each image to illustrate the progressive improvement in segmentation performance. The red mask represents the segmentation output generated at each preprocessing stage.

Qualitative visualization of SC segmentation results from each of the preprocessing stages (obtained after each stage) is shown in Figure 8 for non-compressed and compressed SC cases. The overall impact of individual preprocessing steps is reflected in the figure, which compares the automated results to the manual ground truth for each stage of the processing sequence.

As shown in Figure 8, by visually comparing the intermediate results with the manual segmentation, which clearly indicates that each preprocessing stage is a beneficial factor in reducing segmentation errors, minimizing boundary irregularities, and improving structural continuity. Even for compressed SC images, which initially had deformation and reduced contrast, these solutions from modified learning schemes are visible.

Overall, segmentation accuracy and robustness consistently improved with more advanced preprocessing strategies being applied, while the multi-channel representation tended to yield the highest as well as most stable performance across the dataset.

### 3.3. Statistical Analysis Results

To determine whether the observed improvements were statistically significant, Dice scores were compared between each preprocessing configuration and the baseline model using the Wilcoxon signed-rank test. Comparisons were performed at both the volume level and the slice level, with slices categorized as non-compressed, moderately compressed, and severely compressed (Table 9).

As shown in Table 9, statistically significant improvements at the volume level were limited, likely due to the relatively small number of test volumes and the predominance of non-compressed anatomy within each volume. In contrast, slice-level analysis revealed more pronounced effects of preprocessing. Improvements were generally not significant in non-compressed slices, whereas significant improvements were consistently observed in moderately and severely compressed slices.

The strongest effect was observed for the multi-channel representation, which achieved statistically significant improvements at both the volume and slice levels. N4 correction, NLM denoising, Z-score normalization, augmentation, CLAHE, and LoG filtering showed their greatest benefit in compressed slices, supporting the hypothesis that preprocessing is most valuable in regions affected by SC compression, boundary loss, and anatomical deformation.

## 4. Discussion

Results show that preprocessing improved both image quality and robust segmentation for compressed SC MRI. Across all sites, bias field correction was performed to reduce intra-cord intensity variability, improve intensity homogeneity and minimize scanner-related bias. This step does not directly improve boundary contrast but deals with low-frequency intensity inhomogeneities and offers a more stable input for further processing.

NLM denoising resulted in improved cord–CSF contrast without compromising SC signal magnitude—as suggested by steady SNR and increased CNR values, particularly in datasets with poorer baseline image quality. This behaviour also matched SC MRI, in which the small structure size and weak boundaries are sensitive to noise. The minimal impact on the high-SNR site also implies that NLM adapts itself to data quality rather than over-smoothing already clean input.

While not directly enhancing local contrast or noise properties, Z-score normalization succeeded in equalizing intensity distributions among subjects and sites. Given the initial cropping around the SC, this step was focused on mitigating scanning-dependent scaling effects rather than biological variability.

In addition to the redistribution of intensity values between SC and CSF, CLAHE improved local contrast, leading to an overall change for increasing CNR in all sites. NLM was used to destroy noise and added little diffusion along local intensity gradients. On the other hand, CLAHE did not contribute to amplifying noise excessively and enhanced low-intensity areas that improved local contrast more effectively, especially after applying denoising. Also, edge enhancement using LoGs improved median definition by emphasizing high-frequency components of anatomical edges. The consistently high values for the EPI suggest that LoG bolsters SC boundaries without compromising structural integrity. CLAHE focused on increasing the contrast for pixels in a specified region, and LoG focused on sharpening the edges of objects, resulting in improved segmentation performance by combining these complementary processes.

The performance gain on the multi-channel input hardware was due to complementary information provided by different representations of the same image. The network received simultaneous access to intensity, local contrast and boundary cues by passing the original image along with CLAHE and LoG -enhanced versions as part of a composite input. This reduced the need for the model to learn these features from one shot internally and instead exposed them explicitly, useful in areas of compression where contrast was reduced, and there are no clearly defined boundaries. Consequently, the model can more effectively localize borders of the SC and reduce segmentation error under different imaging conditions. Ultimately, this is reflected in improved robustness and a reduction in extreme failure cases.

The preprocessing steps demonstrated a cumulative effect on segmentation performance. Although individual preprocessing components produced incremental improvements, no single technique was sufficient to fully address the challenges introduced by SC compression. The improvements observed following intensity normalization and data augmentation suggest that reducing inter-subject intensity variability and increasing exposure to anatomical variation improve model generalization across heterogeneous clinical datasets.

The largest performance gains were achieved through the boundary-focused preprocessing strategy, including local contrast enhancement, edge enhancement, and the multi-channel representation. These techniques were specifically designed to compensate for the loss of boundary information that frequently occurs in compressed SC regions, where CSF boundaries become indistinct or completely absent. By providing complementary intensity, contrast, and edge information, the multi-channel framework enabled more accurate delineation of SC boundaries under challenging pathological conditions.

The slice-level analysis suggests that the primary benefit of preprocessing arises in compressed regions rather than in normal-appearing SC. This observation supports the hypothesis that the major challenge in cervical myelopathy is not the segmentation of normal anatomy, but the loss of boundary visibility and anatomical distortion introduced by compression. Consequently, preprocessing techniques that improve intensity homogeneity, local contrast, and boundary representation appear to address the specific image degradations associated with pathological SC compression.

The superior performance of the multi-channel representation further supports this interpretation. By combining complementary intensity, contrast-enhanced, and edge-enhanced representations, the network receives richer structural information than is available from the original image alone. Nevertheless, segmentation performance remained lower in severely compressed slices than in non-compressed slices, indicating that preprocessing alone cannot fully compensate for the loss of anatomical information caused by advanced compression. Future work may therefore benefit from incorporating compression-aware segmentation strategies specifically designed for severely deformed SC anatomy.

The statistical analysis provides additional evidence that the benefits of preprocessing are not uniformly distributed across all anatomical regions. While most preprocessing configurations did not produce statistically significant improvements at the volume level, significant improvements were observed in moderately and severely compressed slices. This finding suggests that volume-level Dice scores may underestimate the contribution of preprocessing because a large proportion of slices exhibit relatively normal SC morphology and are already segmented accurately by the baseline model.

In contrast, compressed slices represent the most challenging regions for automated segmentation due to reduced SC-to-surrounding tissue contrast, boundary ambiguity, and anatomical deformation. The significant improvements observed in these regions indicate that the proposed preprocessing strategies specifically address the image degradations associated with SC compression. The multi-channel representation demonstrated the greatest and most consistent improvements, supporting the hypothesis that combining complementary intensity, contrast, and edge-enhanced representations provides the network with more discriminative information for delineating compressed SC boundaries.

This study has several limitations. First, segmentation performance was evaluated using a single network architecture, as the primary objective was to assess improvements achieved through preprocessing rather than architectural innovation. Although this design isolates the effects of individual preprocessing strategies, it does not account for potential interactions with more complex segmentation models. Second, despite its multi-site nature, the dataset was limited to cervical myelopathy cases and T2-weighted imaging; therefore, the generalizability of the proposed framework to other SC pathologies and imaging sequences requires further investigation. Third, the preprocessing pipeline was evaluated in a modular fashion and did not consider joint optimization of preprocessing and network training. Finally, direct comparison of the reported Dice scores with those of previous studies should be interpreted with caution. The present study used a unique multi-center dataset of patients with cervical myelopathy that has not been evaluated in previous segmentation studies. Moreover, published SC segmentation results have been obtained using datasets that differ substantially in imaging protocols, scanner characteristics, image quality, and the severity of SC compression. Because segmentation performance is strongly influenced by these factors, numerical comparison of Dice scores across different datasets may not provide a fair assessment of method performance. Therefore, the present study focused on the relative improvements achieved by the proposed preprocessing framework under a consistent experimental setting, in which all preprocessing strategies were evaluated using the same dataset, network architecture, and evaluation protocol.

Our future work will also lead to an optimized end-to-end SC MRI analysis pipeline. These include settings for the assessment of state-of-the-art deep learning architectures, applying transfer learning and domain adaptation strategies to improve generalization across sites, and the joint data-dependent optimization of preprocessing and segmentation in unified frameworks. Future studies, for example, will explore how to leverage these automatic segmentations in combination with diffusion MRI and quantitative biomarkers that allow for a more comprehensive assessment of SC pathology and enhance the clinical applicability of automated segmentation.

## 5. Conclusions

This study demonstrates that pathology-aware preprocessing significantly enhanced SC segmentation performance in multi-site cervical spondylotic myelopathy MRI. Instead of implementing a modular approach focusing on generic enhancement, we define a preprocessing sequence where each individual component attempts to correct a particular failure mode associated with intensity inhomogeneity, noise, inter-site variability, or boundary degradation. While every single step provided a marginal benefit, the combination of all steps resulted in a consistent improvement both for accuracy and robustness. In challenging scenarios, the single T2*-weighted scan-based multi-channel representation outperformed others and shows the highest and most stable performance in all subjects. These findings underscore the necessity of structured, task-driven preprocessing for reliable deep learning-based SC analysis and support as a basis for subsequent quantitative and clinical applications.

## Figures and Tables

**Table 1:** Distribution of axial slices across compression categories in the training and test datasets.

Dataset	Non-Compressed (%)	Moderate (%)	Severe (%)
Training	63.8	22.4	13.8
Test	61.9	23.7	14.4

**Table 2:** Experimental design for evaluating the impact of preprocessing techniques.

Experiment ID	Preprocessing Type	Dataset Split	Goal
Baseline	None	Train, Validation, Test	Establishes reference performance
C1-A	N4 Bias Field Correction	Train, Validation, Test	Tests intensity homogeneity impact
C1-B	NLM Denoising	Train, Validation, Test	Tests noise-suppression impact
C2-A	Z-Score Normalization	Train, Validation, Test	Tests cross-site generalization
C2-B	Augmentation	Training only	Tests cross-site generalization
C3-A	CLAHE Histogram Equalization	Train, Validation, Test	Targets low boundary contrast
C3-B	LoG Edge Filtering	Train, Validation, Test	Targets low boundary contrast
C3-C	Multi-Channel	Train, Validation, Test	Evaluates overall pipeline effect

**Table 3:** Effects of N4 bias field correction on SC intensity homogeneity (*n* = 194).

Group	Mean CoV Before	Mean CoV
Site 1	0.121	0.095
Site 2	0.140	0.105
Site 3	0.152	0.119
All	0.138	0.107

**Table 4:** Effect of NLM denoising on SNR and CNR (*n* = 194).

Group	Mean SNR		Mean CNR	
	Before	After	Before	After
Site 1	64.176	64.582	12.231	12.880
Site 2	31.266	31.668	7.144	7.430
Site 3	191.306	191.378	36.106	36.193
All	95.066	95.358	18.421	18.752

**Table 5:** Effect of Z-score normalization on inter-subject intensity variance calculated from mean image intensities across subjects (*n* = 194).

Group	Inter Subject Variation Before (Mean ± SD)	Inter Subject Variation After (Mean ± SD)
Site 1	8844.65 ± 73.67	0 ± 0.99
Site 2	4010.86 ± 53.65	0 ± 0.99
Site 3	3372.71 ± 121.15	0 ± 0.99
All	8597.49 ± 82.46	0 ± 0.99

**Table 6:** Effect of CLAHE on CNR (*n* = 194).

Group	Mean CNR	
	Before	After
Site 1	12.880	15.436
Site 2	7.430	10.582
Site 3	36.193	36.947
All	18.752	21.922

**Table 7:** Effect of LoG on EPI (*n* = 194).

Group	EPI (Mean ± SD)
Site 1	0.78 ± 0.3
Site 2	0.75 ± 0.4
Site 3	0.84 ± 0.2
All	0.79 ± 0.3

**Table 8:** Segmentation results across preprocessing strategies.

Experiment ID	Dice (%)				Precision (%)	Recall (%)
	Volume	Non- Compressed	Moderate Compression	Severe Compression	Volume	Volume
Baseline	81.98	92.88	73.21	49.63	78.96	88.34
C1-A	82.76	92.99	75.03	52.52	78.20	89.61
C1-B	82.97	93.00	75.53	53.17	81.36	86.06
C2-A	83.69	93.11	77.42	56.25	82.12	86.77
C2-B	84.89	93.33	80.25	61.38	83.06	88.04
C3-A	85.06	93.33	80.81	62.04	83.30	88.07
C3-B	85.24	93.44	81.43	63.27	85.89	88.96
C3-C	86.42	93.88	84.62	68.43	87.33	89.85

**Table 9:** Statistical comparison of Dice scores between preprocessing configurations and the baseline model using the Wilcoxon signed-rank test.

Comparison	Volume	Non- Compressed	Moderate Compression	Severe Compression
Baseline vs. N4	0.091	0.212	0.038	0.026
Baseline vs. NLM	0.082	0.185	0.032	0.024
Baseline vs. Z-score	0.104	0.156	0.049	0.033
Baseline vs. Augmentation	0.058	0.084	0.026	0.019
Baseline vs. CLAHE	0.071	0.151	0.047	0.041
Baseline vs. LoG	0.052	0.097	0.044	0.038
Baseline vs. Multi-channel	0.018	0.066	0.021	0.014

Statistically significant differences were defined as *p* < 0.05.

## Data Availability

The datasets analyzed in this study contain sensitive medical information and are subject to ethical and institutional restrictions. Therefore, they are not publicly available. Access to the data may be granted upon reasonable request to the corresponding author and with appropriate institutional approvals. The source code used for data preprocessing, analysis, and model implementation is publicly available on GitHub at: https://github.com/H-Toufani/spinalcord-mri-preprocessing (version v1.0.0) (accessed on 12 May 2026).

## Abbreviations

The following abbreviations are used in this manuscript:

SC	Spinal cord
MRI	Magnetic resonance imaging
CSF	Cerebrospinal fluid
FA	Fractional anisotropy
MD	Mean diffusivity
AD	Axial diffusivity
RD	Radial diffusivity
DTI	Diffusion tensor imaging
ROI	Region of interest
FOV	Field of view
CR	Compression ratio
AP	Anteroposterior
NLM	Non-local means
CoV	Coefficient of variation
SNR	Signal-to-Noise Ratio
CNR	Contrast-to-Noise Ratio
EPI	Edge preservation index
CLAHE	Contrast-limited adaptive histogram equalization
LoG	Laplacian of Gaussian
